# Development of a Silicone Rubber Mold with an Innovative Waterfall Cooling Channel

**DOI:** 10.3390/polym16020256

**Published:** 2024-01-16

**Authors:** Chil-Chyuan Kuo, Pin-Han Lin, Jing-Yan Xu, Zhe-Xhi Lin, Zi-Huan Wang, Zhi-Jun Lai, Song-Hua Huang

**Affiliations:** 1 Department of Mechanical Engineering, Ming Chi University of Technology, No. 84, Gungjuan Road, New Taipei City 24301, Taiwan; 2 Research Center for Intelligent Medical Devices, Ming Chi University of Technology, No. 84, Gungjuan Road, New Taipei City 24301, Taiwan; 3 Department of Mechanical Engineering, Chang Gung University, No. 259, Wenhua 1st Road, Guishan District, Taoyuan City 33302, Taiwan; 4 Center for Reliability Engineering, Ming Chi University of Technology, No. 84, Gungjuan Road, Taishan District, New Taipei City 24301, Taiwan; 5 Li-Yin Technology Co., Ltd., No. 37, Lane 151, Section 1, Zhongxing Road, Wugu District, New Taipei City 241, Taiwan

**Keywords:** silicone rubber mold, conformal cooling channel, waterfall cooling channel, cooling time, simulation

## Abstract

A conformal cooling channel (CCC) follows the mold core or cavity profile to carry out uniform cooling in the cooling stage. However, the significant pressure drop along the cooling channels is a distinct disadvantage of the CCC. In this study, an innovative waterfall cooling channel (WCC) was proposed and implemented. The WCC cools the injected products via surface contact, replacing the conventional line contact to cool the injected products. The WCC was optimized using numerical simulation software. Silicone rubber molds with two kinds of cooling channels were designed and implemented. The cooling time of the molded part was evaluated using a low-pressure wax injection molding machine. The experimental results of the cooling time of the molded part were compared with the simulation results from numerical simulation software. The results showed that the optimal mesh element count was about 1,550,000 with a mesh size of 1 mm. The simulation software predicted the filling time of the water cup injection-molded product to be approximately 2.008 s. The cooling efficiency for a silicone rubber mold with a WCC is better than that of the silicone rubber mold with a CCC since the core and cavity cooling efficiency is close to 50%. The pressure drop of the WCC is smaller than that of the CCC, which reduces the pressure drop by about 56%. Taking a water cup with a mouth diameter of 70 mm, a height of 60 mm, and a thickness of 2 mm as an example, the experimental results confirmed that the use of the WCC can save the cooling time of the product by about 265 s compared with the CCC. This shows how a WCC can increase cooling efficiency by approximately 17.47%.

## 1. Introduction

A conformal cooling channel (CCC) [1] refers to a specific category of the cooling system designed for dies or molds used in various manufacturing processes, such as blow molding [2], metal forming [3], plastic injection molding [4], die casting [5], and metal injection molding [6]. The feature of CCC is to optimize heat dissipation during the cooling stage of the various manufacturing processes. CCC is known for its ability to conform to the shape of the molded product manufactured.

Vargas-Isaza et al. [7] evaluated the cooling efficiency of polymer injection molds with CCC using a numerical simulation. The results showed that a 9.26% reduction in the warpage of the cup-shaped injection molded part was obtained using a CCC compared with the conventional cooling channel. Nguyen et al. [8] used the response surface methodology to determine the optimum CCC shape in an injection mold. The results showed that the temperature distribution of the CCC mold surface was more uniform than that of the conventional cooling channel. Minh et al. [9] optimized the cooling channel in injection molds using Taguchi-integrated principal component analysis. It was found that CCC shows an average temperature peak at 58.78 °C. Choi et al. [10] used the biomimetic engineering method to design the CCC. The results showed that the pressure loss was reduced by about 10 times. In addition, the temperature deviation of the part was improved by approximately 46% using the CCC compared to the part using a conventional cooling channel. Torres-Alba et al. [11] proposed an innovative CCC system that is highly susceptible to warping. The results indicated that the cycle time decreased by about 66%. The residual stress was also reduced by about 81.88%. Gotlih et al. [12] proposed a method for CCC system selection based on non-dominated sorting. The simulation results revealed that the CCC system provides the lowest warpage and shortest cycle times. Kanbur et al. [13] focused on the metal additive manufacturing of a plastic injection mold insert with a CCC system. The deviations between the printed and design parameters were less than 5% for the circular and tapered channels. Torres-Alba et al. [14] presented a hybrid cooling model based on the CCC system in combination with mold inserts. The results showed that the CCC system improves the gradient of the temperature map and uniformity by approximately 51.666%. Torres-Alba et al. [15] presented a new CCC for application in optical parts of great thickness, deep cores, and optical requirements. The results showed that the cycle time of the injection molded plastic part by about 32%.

A CCC [16,17,18,19,20,21] is extensively employed in the injection molding process due to its consistent cooling during the cooling stage post-filling. However, a notable drawback of CCC is the considerable pressure drop along the cooling channels. To solve this drawback, this study proposes an innovative waterfall cooling channel (WCC), and a silicone rubber mold incorporating WCC was developed and implemented. Two types of silicone rubber molds were fabricated using silicone rubber. The cooling duration of the molded component was evaluated using a low-pressure wax injection molding machine. A rapid tool with WCC was designed and optimized using Moldex3D simulation software. The cooling time of the silicone rubber mold with WCC and CCC was investigated experimentally. The experimental results of the cooling time of the molded part were compared with the simulation results.

## 2. Experimental Details

**Figure 1:**
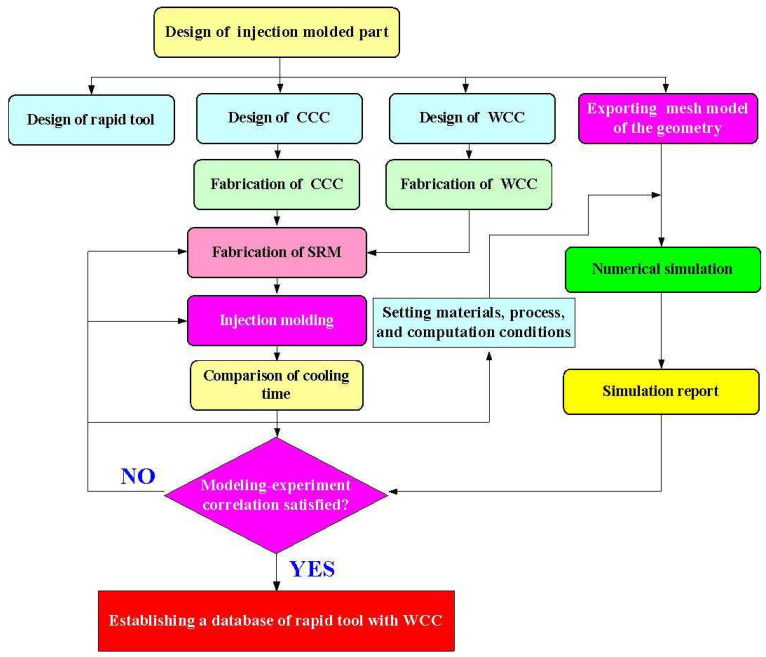
Flowchart of experimental methodology.

**Figure 2:**
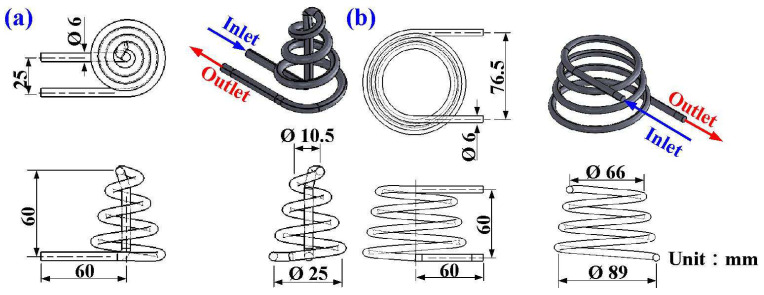
Three-dimensional CAD model and dimensions of the CCC for (**a**) core and (**b**) cavity.

Figure 1 shows the flowchart of the experimental methodology. In this study, a molded part is a water cup featuring a shell thickness of 2 mm, a top diameter of 70 mm, a bottom diameter of 35 mm, and a height of 60 mm. The WCC was optimized using the Moldex3D numerical simulation software. The general procedures of running an injection molding simulation are shown in the figure, which involves pre-processing, analysis setup, and post-processing. Figure 2 shows the 3D CAD model and dimensions of the CCC for the core and cavity. According to the guidelines for the design of CCC, the channel diameter is 6 mm since the wall thickness of the injection molded part is 2 mm. The channel centerline to the mold wall distance is 12 mm since this distance is about two times the channel diameter. The pitch distance between cooling channels is 8 mm since the distance is about three times the channel diameter.

**Figure 3:**
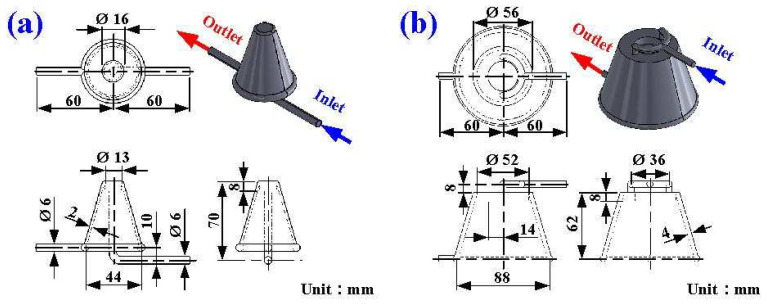
Three-dimensional CAD model and dimensions of the WCC for (**a**) core and (**b**) cavity.

**Figure 4:**
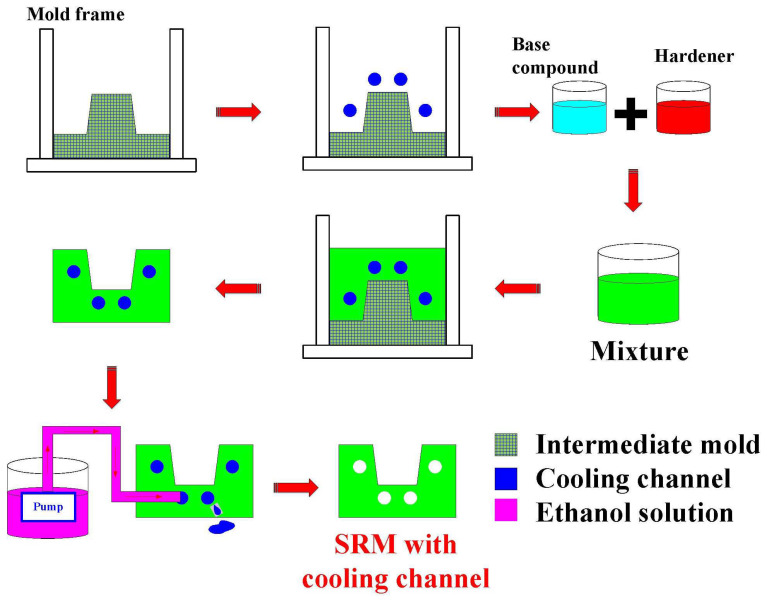
Detailed production flow chart of the silicone rubber mold with a cooling channel.

This study proposed an innovative surface-cooled WCC. The design concept of the WCC includes the following: (a) The cooling liquid flow pattern of the WCC of the cavity insert is fountain flow; (b) The coolant flow path of the WCC of the core insert is series, parallel, and serial connections; (c) The coolant flow path of the WCC of the cavity insert is series, parallel, and series connection; and (d) the WCC of the core and cavity inserts are both designed with water reservoirs to prevent a coolant backflow in the waterfall area. Figure 3 shows the 3D CAD model and dimensions of the WCC for the core and cavity. Figure 4 shows the detailed production flow chart of the silicone rubber mold with a cooling channel. Generally, the three-dimensional printing (3DP) technique offers the capability to produce complex geometries in the form of cooling channels. Thus, a fused deposition modeling machine (Teklink Solution Inc., New Taipei City, Taiwan) [22] was employed to print WCC and CCC using polyvinyl butyral (PVB) filament stocks (Thunder 3D Inc., New Taipei City, Taiwan) [23]. The printing parameters for the PVB cooling channel involve a layer thickness of 0.1 mm, a printing bed temperature of 65 °C, a printing speed of 70 mm/s, and a printing temperature of 200 °C. An industrial alcohol solution was employed to remove the PVB cooling channels inside the silicone rubber mold.

**Figure 5:**
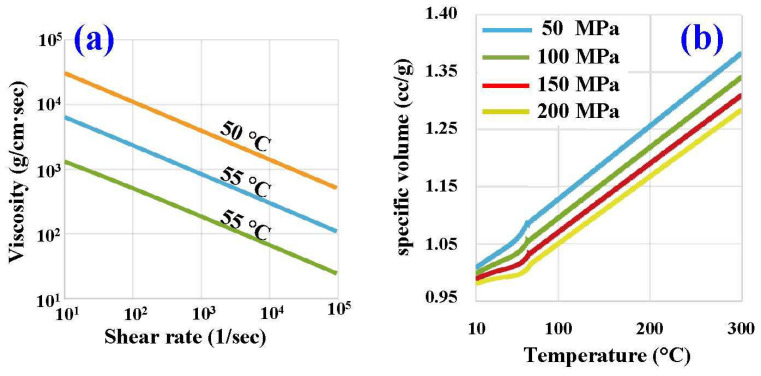
Injection molding simulation conditions of (**a**) viscosity and (**b**) PVT curves.

To understand whether the WCC is better than the conventional CCC, this study used mold flow software to conduct analysis. Figure 5 shows the injection molding simulation conditions of viscosity and pressure–volume–temperature (PVT) curves. Table 1 shows the injection molding simulation conditions. Table 2 shows the properties of the silicone rubber mold. Table 3 shows the properties of the molding material. Wax (K512, Kato Inc., New Taipei City, Taiwan) was used as a molding material because the molded patterns can be employed for investment casting. The silicone rubber (KE-1310ST, Shin Etsu Inc., New Taipei City, Taiwan) and curing agent (CAT-1310S, Shin Etsu Inc., New Taipei City, Taiwan) were mixed in a weight ratio of 10:1 to manufacture a silicone rubber mold (SRM). The mixing process was conducted using a vacuum machine (F-600, Feiling, Inc., New Taipei City, Taiwan) to eliminate air bubbles under vacuum conditions.

**Figure 6:**
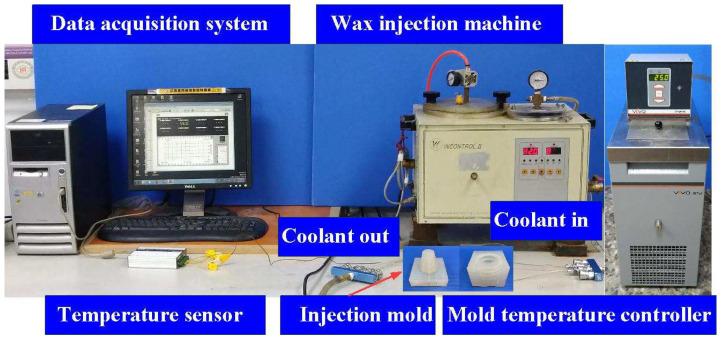
A system for measuring the cooling time of the molded wax pattern.

Figure 6 illustrates the setup employed for measuring the cooling time of the molded wax pattern. The injection molding experiments utilized a low-pressure wax injection molding machine (0660, W&W Inc., Lake Zurich, IL, USA). This configuration included K-type thermocouples (C071009-079, Cheng Tay Inc., New Taipei City, Taiwan) with a measurement sensitivity of ±1 °C, a mold temperature controller (JCM-33A, Shinko Inc., Tokyo, Japan), and a coolant reservoir with a thermo-electric cooler (TEC12706AJ, Caijia Inc., Taipei City, Taiwan). The temperature sensors were strategically placed in the silicone rubber mold cavity, and their other ends were connected to a data acquisition system (MRD-8002L, IDEA System Inc., New Taipei City, Taiwan). The ambient temperature was maintained at 27 °C. The horizontally oriented silicone rubber mold received molten wax at 82 °C into a mold cavity set at 27 °C. The ejection temperature for the molded wax patterns was set at 30 °C through a series of test runs. The inlet coolant temperature was kept at 27 °C. The temperature histories of the molded wax patterns were recorded using temperature sensors.

## 3. Results and Discussion

**Figure 7:**
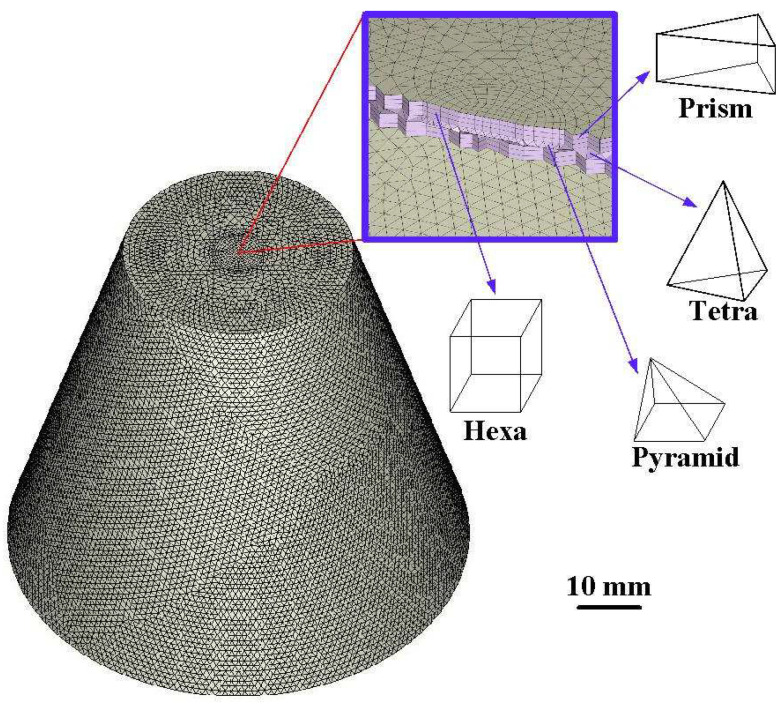
Mesh of the water cup.

**Figure 8:**
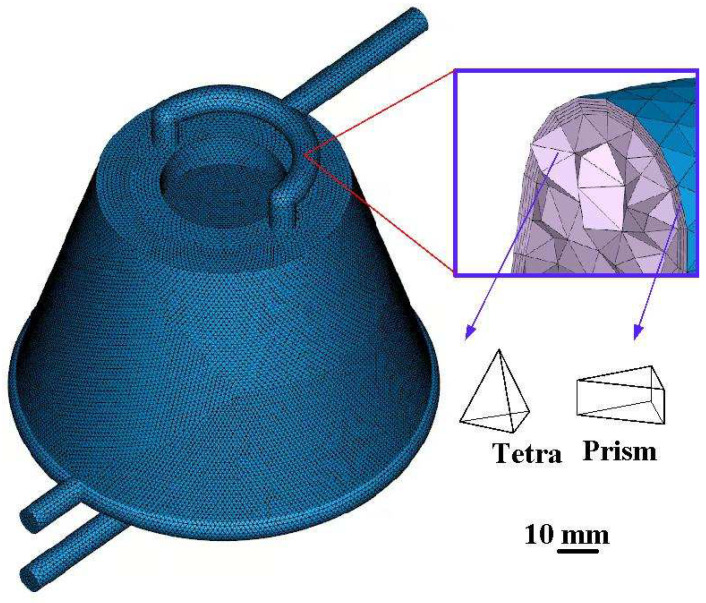
Mesh of the WCC.

A boundary layer mesh (BLM) [24] has several advantages in mold flow simulations, mainly when dealing with flows near solid surfaces. In this study, BLM was used for mold flow simulations because the primary purpose of a BLM is to provide a higher mesh resolution near the walls of the solid surfaces. It is crucial for accurately capturing the boundary layer. In addition, a BLM boundary is compatible with intricate geometric models. The number of BLMs was five in this study. The three-dimensional solid mesh comprises a tetrahedron and prism. Figure 7 shows the mesh of the water cup. The number of boundary layers was 5, and the number of elements was 231,981. The numbers of pyramids, tetrahedrons, prisms, and Hexa were 224, 153,366, 77,719, and 672, respectively. Figure 8 shows the mesh of the WCC. The number of boundary layers was 5, and the number of elements was 671,852. The number of tetrahedrons and prisms was 524,045 and 147,807, respectively.

**Figure 9:**
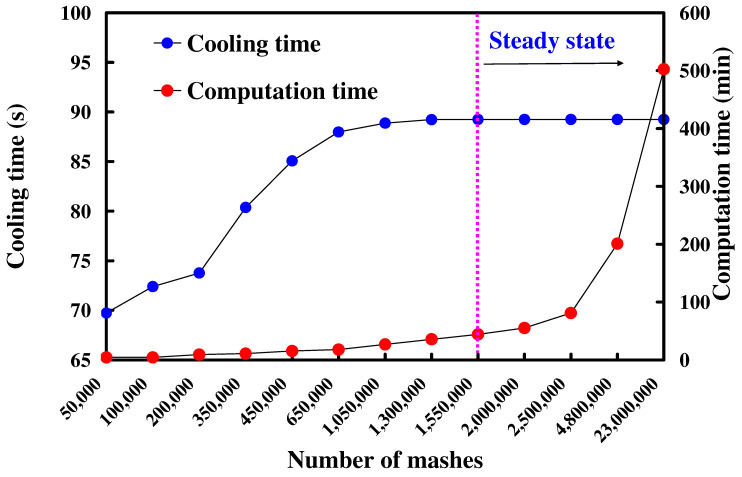
Number of meshes as a function of the computation time and cooling time of the molded part.

**Figure 10:**
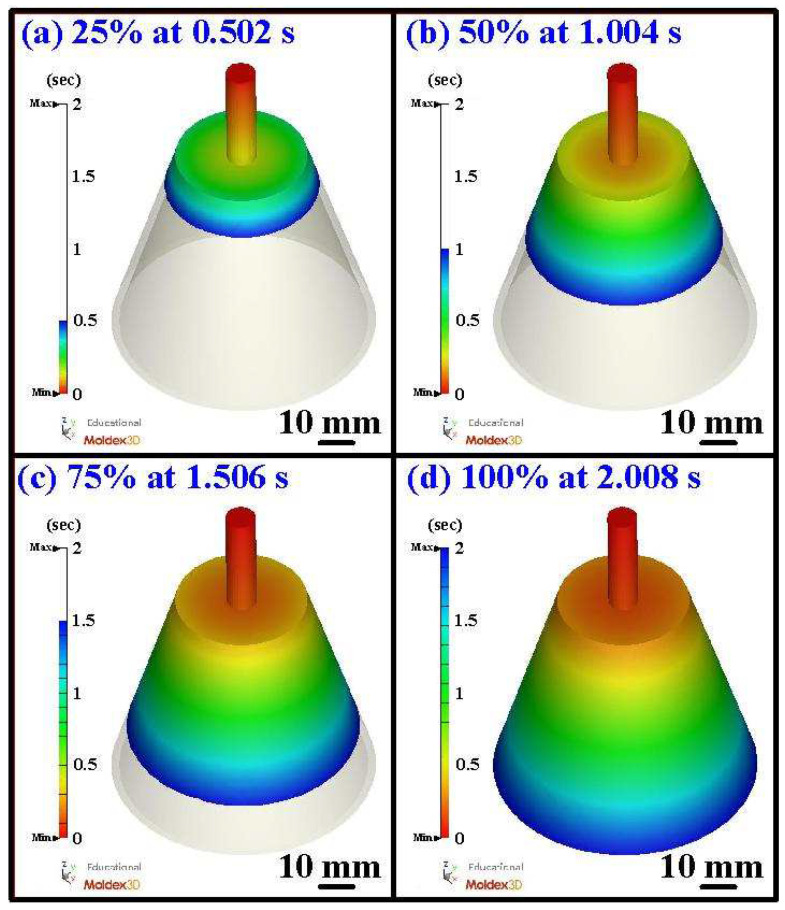
Short shot of the water cup.

Figure 9 shows the number of meshes as a function of the computation time and cooling time of the molded part. According to practical experience, increasing the number of meshes leads to a longer total computation time for the simulation. It becomes evident that the cooling time of the molded part stabilizes when the mesh element count surpasses 1,550,000. The cooling time of the molded part is about 89.24 s. Consequently, a mesh size of approximately 1 mm seems to be the optimal choice, considering both the cooling time of the molded part and the total computation time of the simulation. Figure 10 shows the short shot of the water cup. According to the simulation results, the filling process exhibited a smooth flow, completing in approximately 2.008 s. After the filling was completed, there was no short shot [25] in the molded product, and no meld line [26] or weld line [27] was found. This result also confirms the suitability of the designed filling system [28].

**Figure 11:**
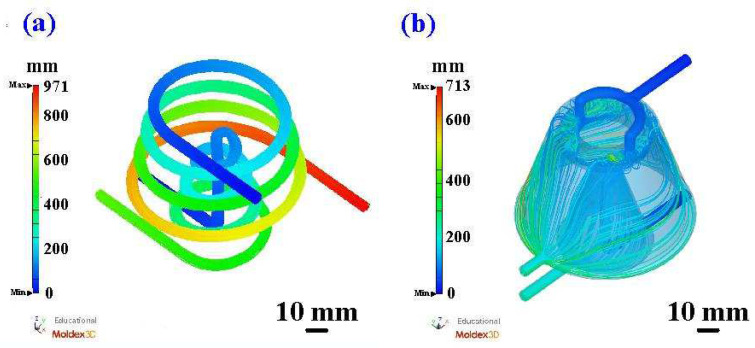
Coolant flow length of silicone rubber mold with (**a**) CCC and (**b**) WCC.

**Figure 12:**
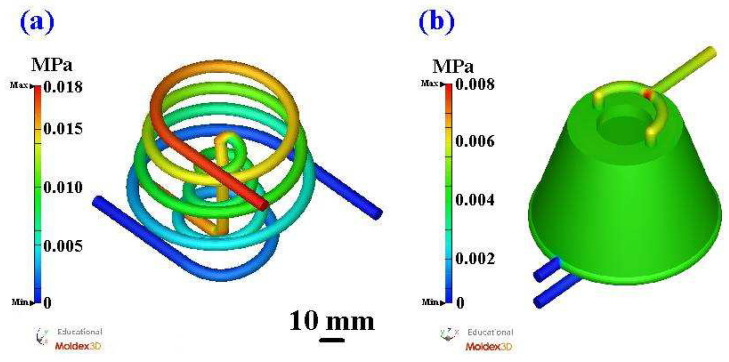
Coolant pressure diagram of silicone rubber mold with (**a**) CCC and (**b**) WCC.

Figure 11 shows the coolant flow length of a silicone rubber mold with the CCC and WCC. Figure 12 shows the coolant pressure diagram of a silicone rubber mold with the CCC and WCC. The flow length of the CCC in the core insert was about 570 mm, and the pressure drop was about 0.017 MPa. The flow length of the CCC in the cavity insert was about 972 mm, and the pressure drop was about 0.018 MPa. The flow length of the WCC in the core insert was about 285 mm, and the pressure drop was about 0.007 MPa. The flow length of the WCC in the cavity insert was about 713 mm, and the pressure drop was about 0.008 MPa. It should be noted that the pressure drop of the WCC was smaller than that of the CCC. The reduction in pressure drop was about 56%. The CCC is a series of water channels, and a greater pressure is required at the water inlet to allow water to flow out from the outlet. However, the WCC is a parallel water channel, which means that water can flow out from the outlet without using greater pressure. If both use the same pressure to enter water, the time for the CCC to reach the water outlet is shorter since the flow direction of the WCC is freer when water enters. Thus, the heat in the silicone rubber mold can be evenly dispersed and discharged. The CCC is connected in series, which cannot dissipate heat like a WCC. Thus, the CCC leads to a significant temperature difference between the water inlet and the water outlet, and the temperature of the cooling water channel at the outlet end is higher.

**Figure 13:**
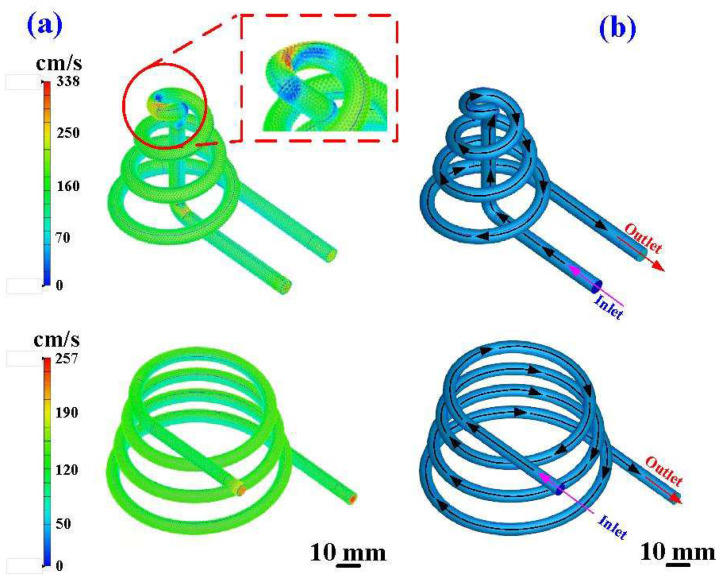
Silicone rubber mold with CCC: (**a**) coolant flow rate and (**b**) schematic diagram of coolant flow.

**Figure 14:**
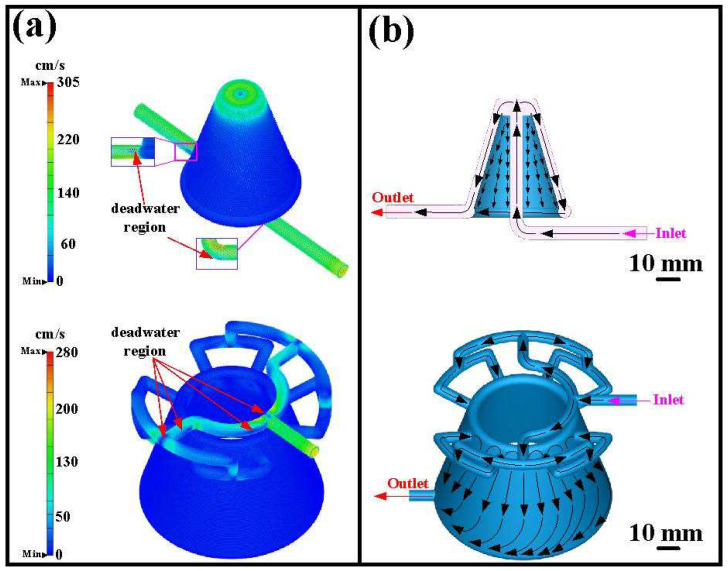
Silicone rubber mold with WCC: (**a**) coolant flow rate and (**b**) schematic diagram of coolant flow.

**Figure 15:**
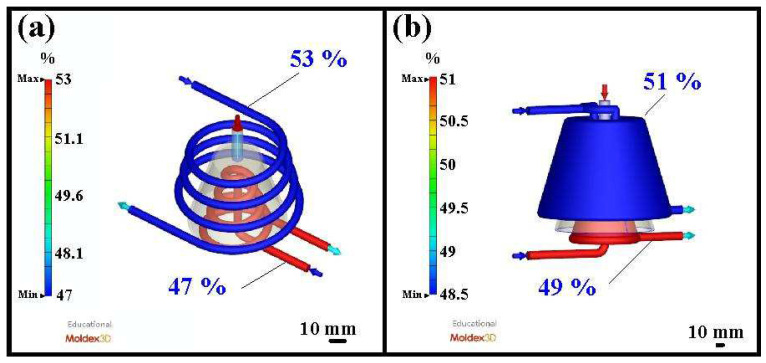
Cooling efficiency of (**a**) silicone rubber mold with CCC and (**b**) silicone rubber mold with WCC.

Figure 13 shows the coolant flow rate and schematic diagram of the coolant flow for the silicone rubber mold with CCC. The results show that the maximum flow velocity of the CCC for the core insert is approximately 338 cm/s, and the maximum flow velocity of the CCC for the cavity insert is approximately 257 cm/s. It should be noted that some dead water regions at the corners were found in the core insert. Figure 14 shows the coolant flow rate and a schematic diagram of the coolant flow for the silicone rubber mold with the WCC. Figure 15 shows the cooling efficiency of the silicone rubber mold with the CCC and the silicone rubber mold with the WCC. As can be seen, the cooling efficiency for a silicone rubber mold with WCC is better than that of the silicone rubber mold with CCC since the core and cavity cooling efficiency is close to 50%.

**Figure 16:**
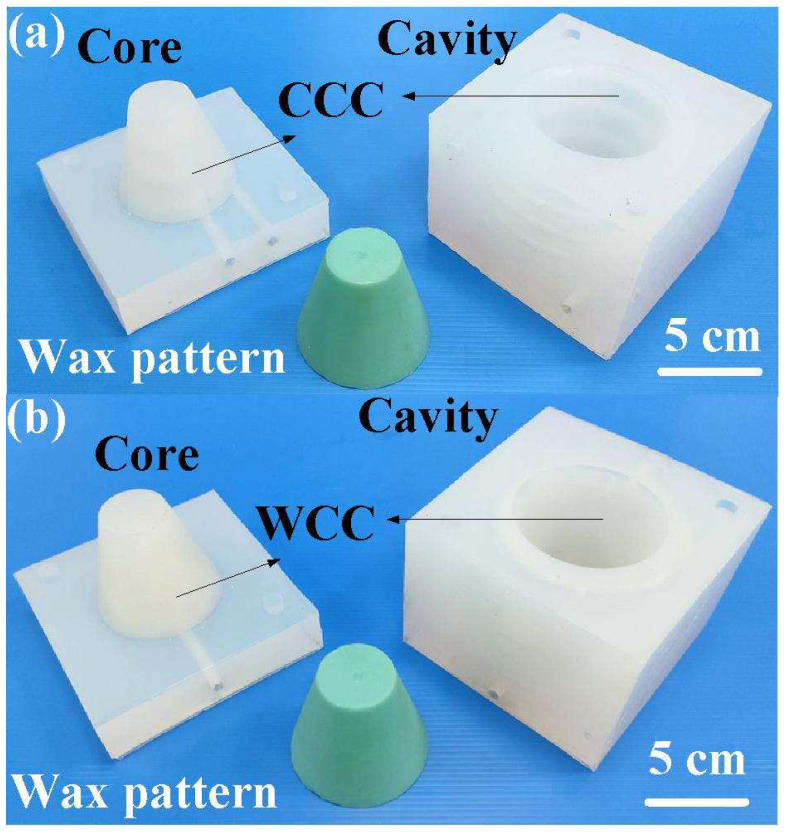
Silicone rubber mold with (**a**) CCC and (**b**) WCC for injection molding.

**Figure 17:**
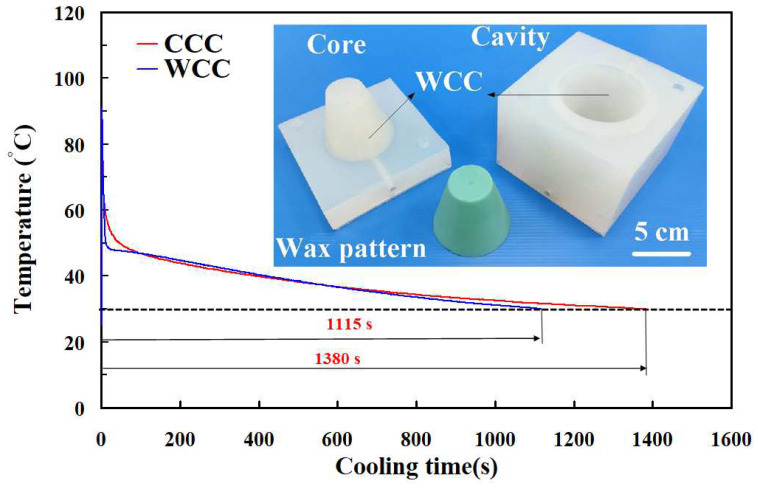
Cooling time of the molded wax pattern for silicone rubber mold with WCC and CCC.

Figure 16 shows the silicone rubber mold with CCC and WCC for injection molding. Figure 17 shows the cooling time of the molded wax pattern for the silicone rubber mold with the WCC and CCC. The cooling time for the water cup injection molded product is approximately 1380 s when utilizing a silicone rubber mold with CCC for low-pressure injection molding. In contrast, the cooling time was reduced to about 1115 s when employing a silicone rubber mold with WCC for the same molding process. This indicates a noteworthy saving of approximately 265 s, demonstrating an improved cooling efficiency of about 19.2% using the WCC. Additionally, this study identified an advantage of the WCCC in the early cooling stage of injection molding. The cooling rate for the water cup injection-molded product reached approximately 1.688 °C/s with a silicone rubber mold equipped with the WCC for low-pressure injection molding. In comparison, the cooling rate was only about 0.538 °C/s when employing a silicone rubber mold with CCC for the same process. Notably, there was no significant difference in the cooling rate between a silicone rubber mold with WCCC and a silicone rubber mold with CCC for low-pressure injection molding. Furthermore, five test runs of injection molding were conducted in this study. The cooling time for the water cup injection molded product using a silicone rubber mold with CCC ranged from approximately 1373 s to 1401 s, with an average cooling time of about 1385 s. On the other hand, the cooling time for the water cup injection-molded product using a silicone rubber mold with WCC varied from approximately 1115 s to 1174 s with an average cooling time of about 1143 s. This indicates an average cooling efficiency improvement of approximately 17.47% using a silicone rubber mold equipped with WCC.

However, the cooling efficiency improvement was about 1% when employing a silicone rubber mold with WCC from the numerical simulation. This variance could be attributed to variations in processing parameters between the simulation software and the experimental environment. These distinctions [29,30,31] encompass properties such as the molding material, silicone rubber mold material, and boundary conditions, including viscoelasticity, melting point, specific gravity, specific volume, linear shrinkage, viscosity, specific heat capacity, thermal conductivity, density, elastic modulus, Poisson ratio, coefficient of linear thermal expansion, coolant inlet temperature, coolant outlet temperature, mold temperature, ambient temperature, and ejection temperature.

This research offers valuable insights for designing molds or dies incorporating the WCC. Generally, the production costs associated with utilizing the WCC or CCC in molds or dies, employing techniques such as direct metal laser sintering, atom diffusion additive manufacturing, selective laser sintering, diffusion bonding, direct metal deposition, or electron beam melting, can be prohibitively high. It is worth noting that WCC-equipped molds or dies find applications in rotational molding [32], centrifugal molding [33], powder injection molding [34], plastic injection molding [35], and blow molding [36]. A specific observation is that molds or dies with the WCC, produced through selective laser melting [37], may encounter issues such as cooling water leakage at connection points. A notable advantage, however, is that the WCC’s molds or dies exhibit no cooling water leakage during plastic injection molding when utilizing a one-process fabrication approach with rapid tooling technology [38,39]. This study utilized silicone rubber [40] to produce the injection molds with a WCC and CCC [41]. Additionally, conventional molded steel [42,43,44,45] can be employed to create injection molds with WCC or CCC through metal additive manufacturing. Investigating pressure variations within the injection mold is also a noteworthy research focus. These issues are currently ongoing studies. The outcomes will be presented in subsequent works.

## 4. Conclusions

The main objective of this study is to propose an injection mold with an innovative WCC. The WCC was optimized using Moldex3D simulation software. This study employed low-pressure wax injection molding to evaluate the rapid mold with two kinds of cooling channels. The cooling time of the molded part was investigated and compared with the simulation results using Moldex3D simulation software. The main conclusions from the experimental work in this study are as follows:

1. The findings of this work highlight significant potential applications in the investment casting industry, mainly due to the notable impact of reduced cooling times on the production costs during the mass production of wax patterns.
2. The results showed that the optimal mesh element count was about 1,550,000 with a mesh size of 1 mm. The simulation software predicted the filling time of the water cup injection-molded product to be approximately 2.008 s.
3. The simulation results revealed that the cooling performance of the WCC was better than that of the CCC since the WCC maintains a uniform and steady cooling performance of the wax pattern than the CCC.
4. The pressure drop of the WCC is smaller than that of the CCC. The reduction in the pressure drop is about 56%. In addition, the cooling efficiency of WCC is better than that of the CCC because the core and cavity cooling efficiency is close to 50%.
5. The use of WCC can save the cooling time of the product by about 265 s compared to the CCC. This shows that WCC can increase the cooling efficiency by approximately 17.47%.

## Figures and Tables

**Table 1:** Injection molding simulation conditions.

Parameters	Data
Injection pressure (MPa)	0.06
Filling time (s)	2
Melt temperature (°C)	82
Injection mold temperature (°C)	27
Shot volume (cm^3^)	22.8
Hodling time (s)	0.1
Coolant flow rate (cm^3^/s)	60
Demolding temperature (°C)	35

**Table 2:** Properties of the silicone rubber mold.

Parameters	Data
Density (g/cm^3^)	1.07
Viscocity (CPS)	75,000
Shore hardness D	40
Tensile strengtg (psi)	850
Elongation (%)	340

**Table 3:** Properties of the molding material.

Parameters	Data
Melting point (°C)	80–85
Specific gravity	0.96
Linear shrinkage (%)	0.9–1.0
Poisson ratio	0.17
Penetration	9

## Data Availability

The data and materials are available.
